# Research on Silicone Rubber Sheds of Decay-Like Fractured Composite Insulators Based on Hardness, Hydrophobicity, NMR, and FTIR

**DOI:** 10.3390/polym14163424

**Published:** 2022-08-22

**Authors:** Zhijin Zhang, Guohui Pang, Ming Lu, Chao Gao, Xingliang Jiang

**Affiliations:** 1 State Key Laboratory of Power Transmission Equipment & System Security and New Technology, Chongqing University, Chongqing 400044, China; 2 State Grid Henan Electric Power Research Institute, Zhengzhou 450015, China

**Keywords:** composite insulator, decay-like fracture, silicone rubber shed, aging

## Abstract

The safety and stability of power systems are seriously threatened by the decay-like fracture of composite insulators. This paper analyzes the aging characteristics (physical properties, NMR, and FTIR) of the silicone rubber sheds of the decay-like fractured insulator. The same V-string insulator and a new insulator are used for comparison. The study shows that the sheds’ degradation is concentrated on the side with heavy pollution. The physical properties (appearance, pollution, hardness, and hydrophobicity) of the high voltage end decrease significantly compared to other positions, but there is no direct connection between the physical properties of sheds and the decay-like fracture of the core rod. The severity of aging increases with a decrease in the equivalent transverse relaxation time T_2_. The main chain of the PDMS material was severely damaged at the location of the insulator fracture. NMR and FTIR can well judge the aging degree of silicone rubber housings. However, no definite characteristic quantity can characterize the decay-like fracture. It is challenging to evaluate the decay-like fracture of the silicone rubber shed only by its aging degree.

## 1. Introduction

Composite insulators have been utilized extensively in China’s power systems since the 1980s due to their excellent anti-flashing performance, high strength, lightweight, easy installation and maintenance [1,2,3,4]. Statistics show that 9 million composite insulators have been consumed in China [5]. Silicone rubber composite insulators are subjected to various environmental stresses. More and more faults occur when composite insulators operate for more years. For instance, the entire power system is significantly impacted by the decay-like fracture of the composite insulator [6]. Studies [6,7,8,9] focused on FRP (Fiber Reinforced Plastics) core rods of decay-like fractured insulators. Liang [6] and Lutz [7] discovered that the core rod was corroded, and the sheath had transverse holes. Furthermore, the epoxy resin matrix of the core rod was oxidized, and the content decreased. Wang [8] considered that the mechanical strength of the core rod is significantly diminished by the degradation of the epoxy resin matrix, which is the cause of composite insulator fracture.

Previous studies have concentrated on the aging characteristics of silicone rubber housings, such as electrical properties (flashover voltage, leakage current), physical properties (hardness, hydrophobicity, morphology), and chemical properties (element content, functional groups) [10,11,12,13,14,15,16]. For instance, in the literature [12], the characteristics (particle size, element composition, molecular group, and phase) of the powdered layer of the composite insulator sheds aged for more than ten years under three different environmental stresses are analyzed. The characteristics of high temperature vulcanization (HTV) silicone rubber (SiR) housings of decay-like fractured composite insulators are seldom investigated. Currently, many evaluation methods exist for composite insulators’ aging state; each has advantages and disadvantages, and no unified standard and index exist. The features and mechanism of the composite insulator with decay-like fracture are unclear because of the small number of samples and the difficulty of sampling. This paper discussed the aging state evaluation of silicone rubber sheds of decay-like composite insulators from the traditional and new aging evaluation methods. The decay-like insulator’s hardness, hydrophobicity, nuclear magnetic resonance, and other characteristics are examined in this paper. The research findings provide guidance for field work and serve as a foundation for further investigation into the process that leads to composite insulators’ decay-like fracturing.

## 2. Materials and Methods

### 2.1. Experimental Samples

**Figure 1:**
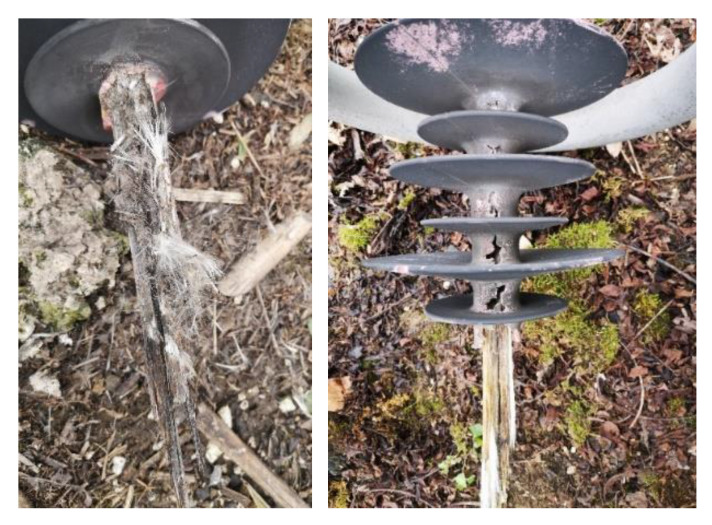
Field pictures of the decay-like fractured insulator.

The test sample came from a B-phase (neutral line) V-shaped composite insulator string of a 500 kV (AC) operating transmission line, and the type was FXBW−500 kV/180 kN. The decay-like fractured composite insulator, unbroken insulator in the same string, and new insulator were tested, respectively, and the corresponding numbers were 1#, 2#, and 3#. For the convenience of description, the test sheds of the grounding side, middle, and high voltage side of 1# are numbered as A, B, and C, respectively. The test sheds of 2# are numbered as D, E, and F, respectively. The test shed of the high voltage side of 3# is numbered as G. These were initial samples with contamination. The detailed fracture, interface failure phenomena, and holes on the sheath are shown in Figure 1.

### 2.2. Experiment Method

- Appearance inspection mainly checks whether the composite insulator has apparent defects, such as color differences, cracks, chalking, and other features. Appearance inspection is mainly to make a basic judgment on the deterioration of insulators;
- Contamination Degree: According to IEC 60507 [17] and IEC/TS 60815−1 [18], the equivalent salt deposit density (ESDD) and non-soluble deposit density (NSDD) of samples were measured through several steps, such as stirring and standing, pollution liquid conductivity test, filtration, drying, and weighing after pollution sampling;
- Hardness: LX−A shore hardness tester (Beijing Time−Top Technology Co., Ltd., Beijing, China) was employed to measure the sample hardness according to ISO 48−4 [19]. The pressure foot is perpendicular to the sample surface, and the pressing speed is not more than 3.2 mm/s. The spring test force should be maintained for 3 s before reading. For the same sample, measure 6 points from the high voltage end to the low voltage end in turn, and take the average value as the hardness value of the sample;
- Hydrophobicity: The spray method was employed according to IEC/TS 62073 and STRI Guide [20,21]. The test process is as follows: firstly, the kettle is used to spray water on the insulator surface, and then the hydrophobic classification (HC) level is determined by observing the surface water droplet state through visual observation;
- The static contact angle method can objectively and accurately measure the hydrophobic state of the material surface. Contact angle refers to the angle between the tangent at the junction of water droplets and sample surface. The static contact angle was measured using the SDC−100 Surface Energy Meter (Ningbo Pres Instrument Technology Co., Ltd., Zhejiang, China). The sessile drop method was employed to measure the static contact angles. The sample should be in a horizontal state when measuring, and then the 10 µL deionized water was dropped on the sample surface. The circular fitting method was used to take the average value of the static contact angles of the left and right ends of the water bead. The same sample was randomly selected 6 to 9 points, and the average arithmetic value was taken;
- Nuclear Magnetic Resonance (NMR): NMR means that under the external magnetic field, the nuclear magnetic moment of matter atom undergoes energy level splitting, and the absorbed energy undergoes energy level transition. NMR detects the physical and chemical properties of the material by applying a pulsed magnetic field to the material and measuring the pulse reflected wave signal [22]. The low field nuclear magnetic resonance measurement platform [23] was employed for the nuclear magnetic detection of samples. Experimental equipment mainly includes a Kea2 nuclear magnetic resonance spectrometer (Magritek, Wellington, New Zealand), a Radio Frequency power amplifier (BT00500 ALPHA-SA, Tomco, Stepney, Australia), a duplexer module, a computer, and a unilateral nuclear magnetic resonance sensor. The system parameters of the magnetic resonance analyzer were set up. For each sample, the CPMG pulse was continuously measured three times, and the transverse relaxation time T and peak area of each peak were retrieved [24,25];
- FTIR (Fourier transform infrared spectroscopy): Attenuated total reflection Fourier transform infrared spectroscopy (ATR FTIR) is a common surface structure analysis technology. To find the corresponding relationship between interferogram and spectrum, a Nicolet iS50 Fourier transform infrared spectrometer (Thermo Fisher Scientific Co., Ltd., Waltham, MA, USA) was used to measure the infrared spectrum of samples, and the wavenumber range was 400−4000 cm⁻^1^.

## 3. Results and Discussion

### 3.1. Physical Characteristics

#### 3.1.1. Appearances

**Figure 2:**
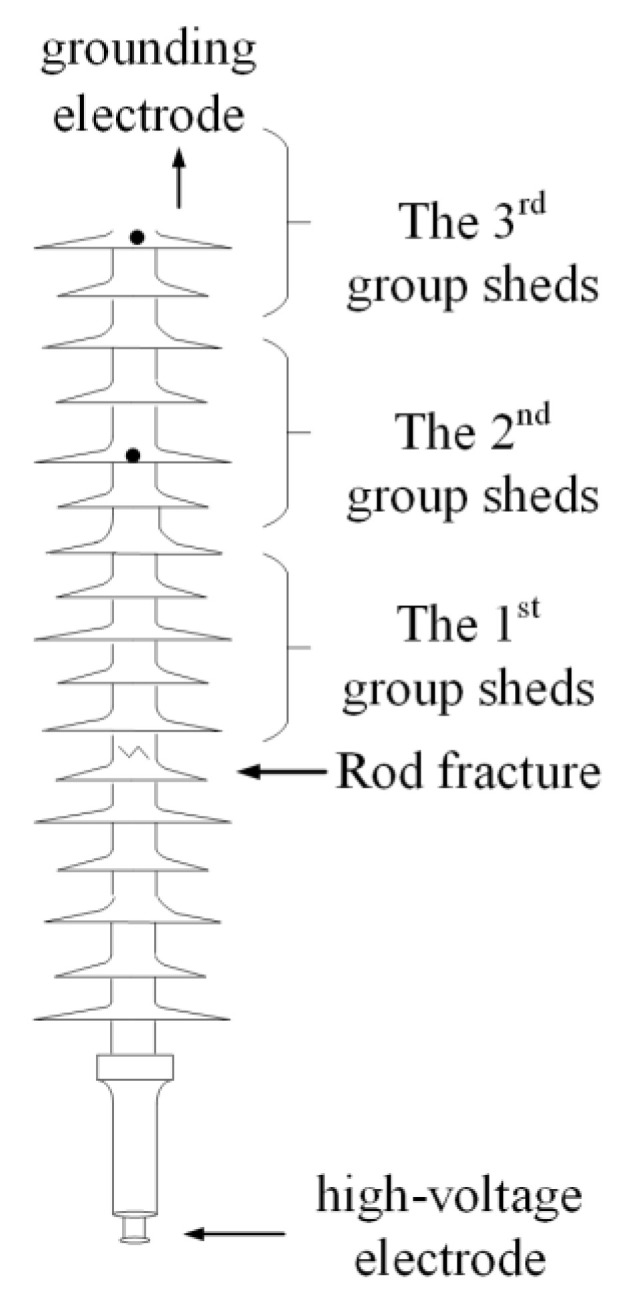
Fracture diagram of composite insulator.

**Figure 3:**
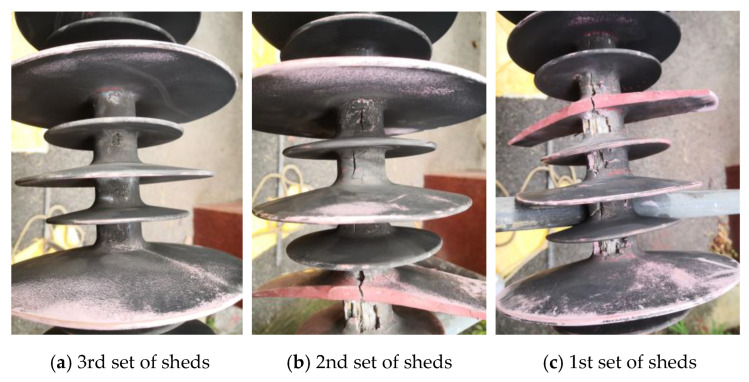
The appearance of 1# insulator (leeward side).

**Figure 4:**
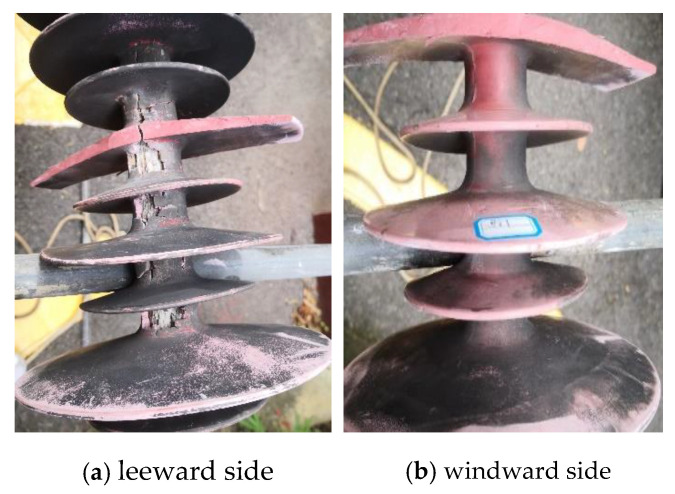
The appearance of the wing/leeward side in the 1st group sheds of the 1# insulator.

**Figure 5:**
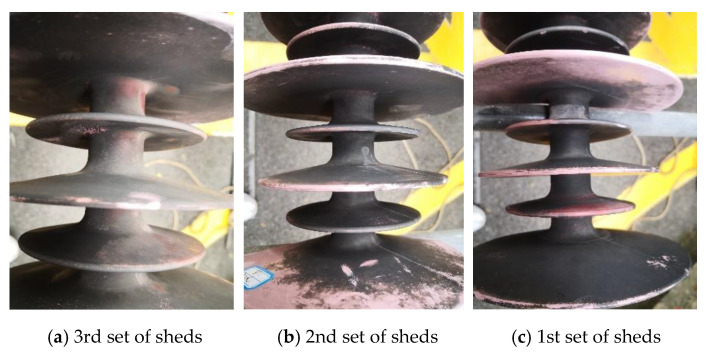
The appearance of 2# insulator (leeward side).

The diagram of the fractured composite insulator is shown in Figure 2. The appearances of decay-like fractured insulators are shown in Figure 3 and Figure 4, and Figure 5 shows the appearance of 2# insulator.

Figure 3 and Figure 4 indicate that when the sheath goes closer to the high voltage end, the breaking worsens, and there are many cracks on the surface. The sheath’s degradation is concentrated on the side with heavy pollution, with no notable occurrence on the other side. Along the direction of the grounding end, the degree of degradation diminishes. As a result, sheath deterioration is strongly linked to leakage current, heating generated by leakage current, and other relevant factors on the leeward side.

Figure 3 and Figure 5 show that the core rod of the decay-like fractured insulator is severely damaged. The core rod’s macroscopic section is rough and like decayed wood. The interface between the glass fiber and epoxy resin matrix is separated, and the interface close to the fracture is invalid. The shed colors are seriously faded. No cracks or fractures are visible on the surface of 2# composite insulator. However, sheds can be visually observed with faded color, chalking, and other deterioration phenomena from 2#.

#### 3.1.2. Pollution

The ESDD and NSDD results of different insulator parts are shown in Table 1.

Table 1 shows that the top surfaces of insulators are prone to accumulate pollution in a natural environment since their contamination degrees (ESDD and NSDD) are higher than those of the bottom surfaces. The bottom surface pollution characteristics at the corresponding positions of the two insulators are almost equal (except NSDD**_bottom_** of A). ESDD_top_ at both ends of 1# is higher than 2#, while NSDD_top_ (and NSDD) is the opposite. The ESDD in the middle part of the two composite insulators is approximate. The ESDD value of C is higher than that of F. The formation of decay-like may be related to contamination. Surface contamination dissolves in water in wet weather, increasing surface conductivity, and then the leakage current increases, damaging the silicone rubber and internal core rod. The primary influencing variables of insulator pollution are air particle characteristics, meteorological conditions, and insulator structures [26,27,28].

#### 3.1.3. Hardness

The hardness (Shore A) of 7 samples before and after boiling is shown in Table 2. The hardness before boiling is K1, and the hardness after boiling is K2.

The hardness of various positions decreases after boiling, as shown in Table 2, and the changes are within 10%. Compared to the hardness of the middle part and low voltage sides, the high voltage side has the highest hardness. The high voltage sides of 1# and 2# have hardness values of 73.2 and 73.4, respectively, indicating no direct connection between silicone rubber hardness and decay-like fracture of the core rod. The hardness of 1# and 2# samples are close to each other, while the hardness of the high voltage side of 3# is 64.8 after boiling, suggesting that insulators become harder with aging. The positions where the relative hardness change (K1−K2)/K1 is the most in the two insulators are the middle (6.07%) and the low voltage side (4.32%), respectively. The position with the smallest change in the decay-like insulator is the high voltage side (2.92%).

Following NMR and FTIR detection, it can be seen that the silicone rubber main chain at the high voltage end is seriously broken. The chemical bonds Si−O and Si−CH_3_ in Polydimethylsiloxane (PDMS) are broken, and Si precipitation increases the silicone rubber’s surface hardness. The electric field intensity at the high voltage end is the highest, and the higher the voltage is, the more likely the electric field is to be distorted. A distorted electric field is prone to generating electrical discharge and serious break of chemical bonds Si−O and Si−CH_3_ [29,30]. Compared with the findings of the pollution test, it is found that the more pollution accumulation, the greater the hardness and the more severe the aging.

#### 3.1.4. Hydrophobicity

**Figure 6:**
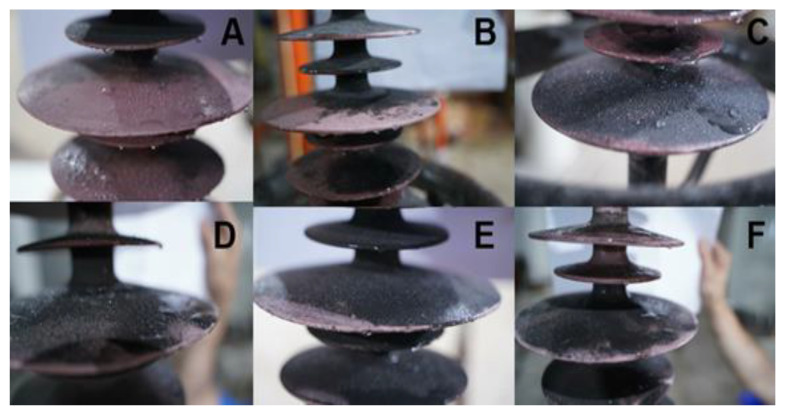
Hydrophobic Classification (HC) measurement of samples (**A**−**F**).

The hydrophobicity of samples A–F was shown in Figure 6 by the spray method.

As seen in Figure 6, most of the water droplets on A, C, D, and F surfaces are small, and some are large. The HC values of A, C, D, and F are considered HC2 according to IEC/TS 62073. The water droplets on the surfaces of B and E are fine, and the HC values of B and E are judged to be HC1. After a long-term operation, the two insulators still maintain good hydrophobicity.

Static contact angles of samples before and after cleaning were examined according to the method described in Section 2.2. The second test was performed with degreased cotton and ethanol to clean the contaminated silicone rubber. The results are shown in Table 3.

Table 3 shows that θ_AVG_ and θ_MIN_ of A, B, C, D, F, and G are all greater than 90°, indicating the samples’ high hydrophobicity. The static contact angle of the polluted insulator is higher than that of the clean insulator. The contamination layer of the composite insulator can obtain hydrophobic substances from the silicone rubber housings so that the contamination layer with high surface tension also shows certain hydrophobic properties. That is, the contamination composite insulator has hydrophobic migration characteristics [31,32].

The static contact angles in the high voltage side, the grounding side, and the middle of the decay-like fractured insulator increase orderly. The static contact angle θ of the high voltage side is larger than that of the grounding side within the same insulator, which is consistent with the above spray method.

From Table 3, it can be shown that after cleaning, F has a larger static contact angle than C. Therefore, the hydrophobicity and the loss of hydrophobicity of C are worse than that of F. Since the high voltage end of C has a decay-like fracture, it may be assumed that there is some relationship between decay-like fracture and hydrophobicity. The worse the hydrophobicity is, the more serious the aging.

The data above show that during operation, the hydrophobicity of the high voltage side of the same insulator is worse than that of various portions. After the two insulators’ operation, the high voltage ends’ static contact angles are still higher than the new insulator’s. The reason may be that the running insulator contamination entered the silicone rubber surface, which enhanced its hydrophobicity, and the sample was not fully dried before the hydrophobicity test. However, C has the worst hydrophobicity among the two operating insulators. The C−H bond in −CH_3_ on the silicone rubber surface is broken by the electrical field strength of the high voltage end, creating the hydrophilic group −OH by the hydrolysis reaction and decreasing the hydrophobicity [33,34]. Due to the high voltage end’s severe aging, methyl levels decline, diminishing the polarity and hydrophobicity of silicone rubber materials, which FTIR can verify.

### 3.2. Chemical Characteristics

#### 3.2.1. NMR Analysis

**Figure 7:**
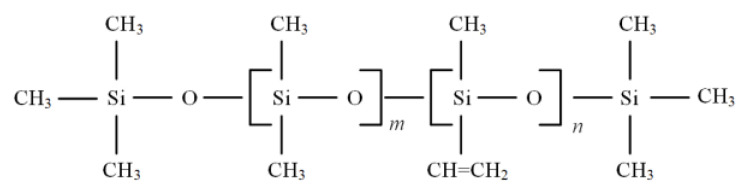
Molecular Formula of PDMS (*m* >> *n*, *m* = 5000–10,000, *n* = 10–20).

PDMS is the main component of silicone rubber, and its molecular formula is shown in Figure 7. Covalent bonds combine each atom in the molecule, and the bond and force are weak, which makes the macromolecules of organic materials easy to break. A certain proportion of carbon black and aluminum trihydrate (ATH filler) is added to materials to increase their mechanical strength and high temperature resistance. NMR experiments were carried out on each insulator sample, and the measured Carr–Purcell–Meiboom–Gill (CPMG) echo signal was inverted using the inverse Laplace transform method to extract the characteristic quantity that can reflect the aging state of the composite insulator, namely the equivalent transverse relaxation time T_2_. The T_2_ is obtained by measuring the state of atom H.

Affected by a harsh external environment or corona discharge, the silicone rubber composite insulator is aging, which causes some groups connected with Si atoms in the main chain to fall off, the number of H atoms decreases, or the state of H atoms in the groups changes. Nuclear magnetic resonance technology uses the magnetic resonance characteristics of the H atomic nucleus to study the properties and environment of the H atomic nucleus in the material and to analyze its molecular structure [23].

NMR experiments were carried out on 7 shed samples of A−G, and the measured CPMG echo signal was inversed using the inverse Laplace transform Method. During the aging process, the methyl group attached to Si broke, and the H atom was separated with the changed state. The distance between H atoms decreases as T_2_ decreases. The simulation of the H atom’s changing state can be used to evaluate aging characteristics. The T_2_ of insulators was extracted as the characteristic value that can reflect the aging state of composite insulators. 

**Figure 8:**
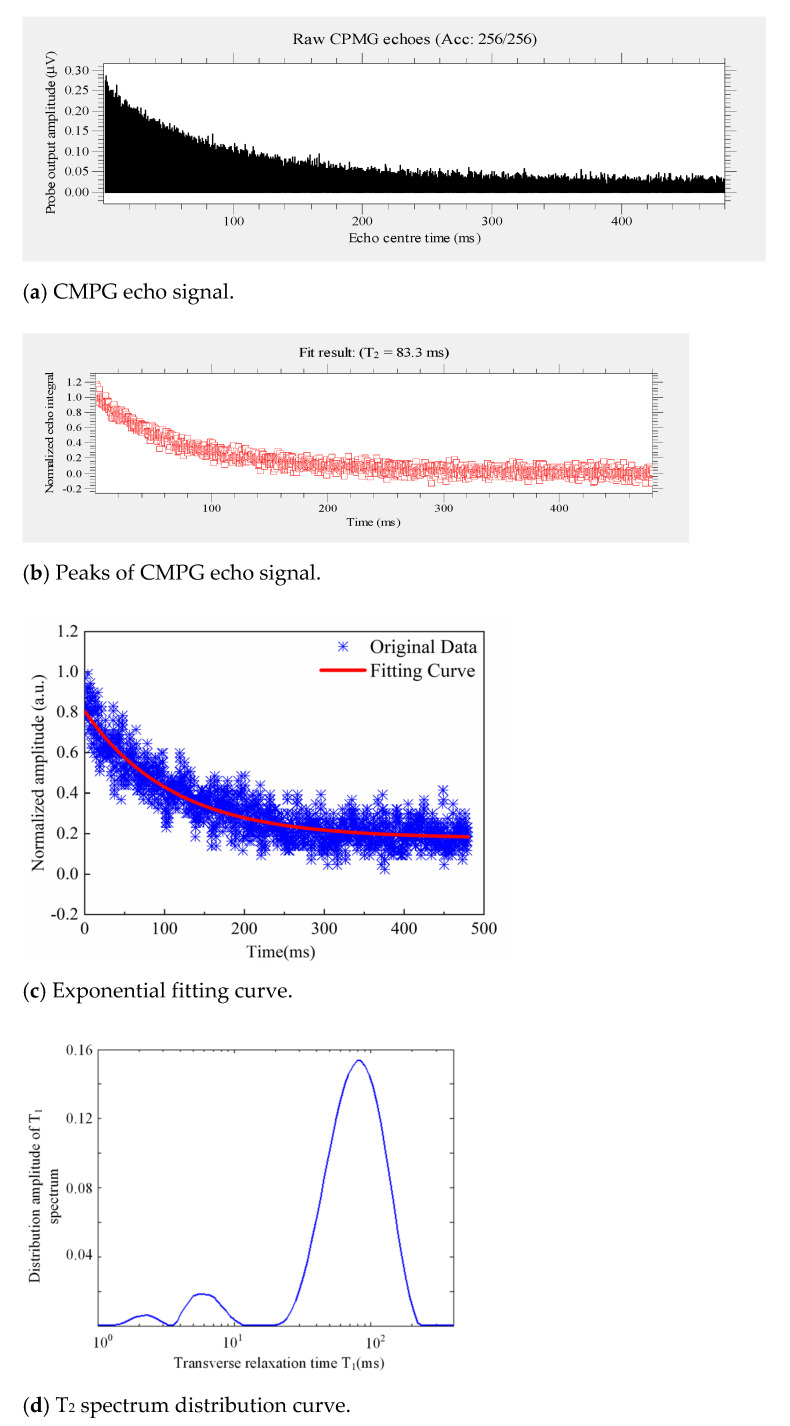
The extraction process of equivalent transverse relaxation time T_2_.

According to the above experimental method, the process is shown in Figure 8 [23,24,25]. The echo time is 120 µs, and the number of echoes is 2000.

The T_2_ of each sample was measured and compared with the average value, as shown in Table 4.

As illustrated average values of different parts of the decay-like insulator in Table 4, the relaxation time of the high voltage end is the shortest, and the relaxation time of the grounding end and the middle are close. The relaxation time of the high voltage end of 2# insulator is also the shortest, followed by the relaxation time of the grounding end.

According to the average values in the same part of different insulators, the relaxation time of the high voltage end of 1# insulator is the shortest at 52.7898 s. The relaxation time at the high voltage end of 2# insulator is 56.1363 s, slightly exceeding 1# insulator. The severity of aging increases with a decrease in T_2_. Compared with 3# insulator, 2# insulator has a shorter equivalent relaxation time and more severe aging.

The values of 1# insulator show that the T_2_ is variable due to contamination. The influence of pollution on the aging degree is uncertain.

Table 4 indicates that the aging degree of the high voltage end is higher than that of other positions. The positions with heavy pollution, high hardness, and poor hydrophobicity generally have severe aging degrees. The aging of the high voltage end of 1# insulator is the most serious, which indicates that aging is closely related to decay-like fracture.

#### 3.2.2. FTIR Analysis

**Figure 9:**
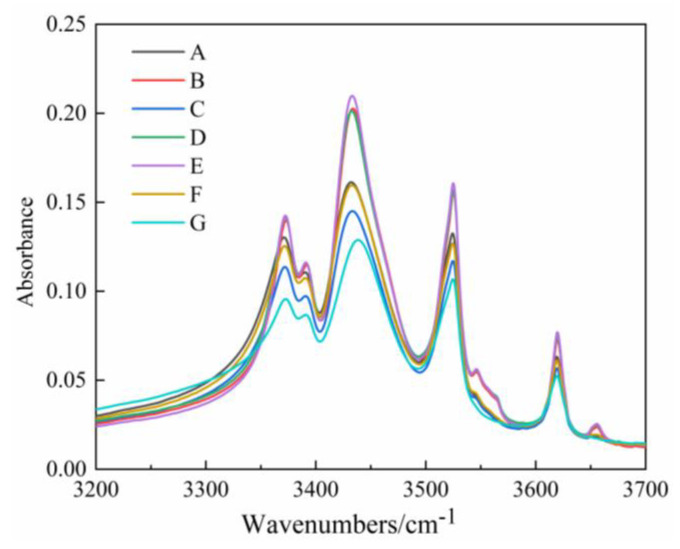
Infrared spectra of 3200–3700 cm^−1^ ranges (−OH).

The main component of silicone rubber is PDMS, mainly composed of the main chain Si−O−Si structure and the side chain Si−CH_3_. The −OH groups are hydrophilic, decreasing hydrophobicity on the silicone rubber surface [29]. FTIR was employed to investigate the functional groups of the silicone rubber samples. The corresponding wavenumbers of different functional groups are shown in Table 5 [35,36]. Samples of A–G (after cleaning) were analyzed and compared by FTIR, as shown in Figure 9.

The −OH functional group causes four peaks at 3200–3700 cm^−1^ in the infrared spectrum. As depicted in Figure 9, −OH content: E>B>D>A>F>C>G, which was consistent with the static contact angle. That is, −OH content is high when the static contact angle is large. The highest and lowest −OH contents on the decay-like fractured insulator are at the middle and high voltage side, respectively, and so is 2# insulator. It can be seen from the two running insulators that −OH content at the corresponding positions is 2#>1#.

Figure 9 shows that the new insulator has the lowest peak value, meaning that its −OH content is the lowest, which is consistent with the hydrophobicity test. Two processes may affect the –OH: (i) Hydrolysis of silicone rubber catalyzed by water, forming hydrophilic silanol groups (Si−OH) and silanol (Si−CH_2_−OH) on the material surface; (ii) The ATH filler inside the sample continuously migrates to the surface and reacts with nitric acid, resulting in the loss of –OH functional groups and the continuous deposition of aluminum (Al) element. At the same time, the migration process of ATH filler will lead to an increase in internal porosity and a decrease in tracking resistance. The increase of internal –OH indicates that (ii) is greater than (i) on the surface.

When the composite insulator works in a high temperature and high heat environment for a long time, water acts as a catalyst to make the silicone rubber hydrolysate, and free radicals are bound. There is more −OH at the high voltage end, and the hydrolysis reaction is severe. The increase of hydrophilic group −OH leads to the weakening of hydrophobicity, and the reasons for the weak hydrophobicity at the high voltage end are analyzed from the chemical properties.

**Figure 10:**
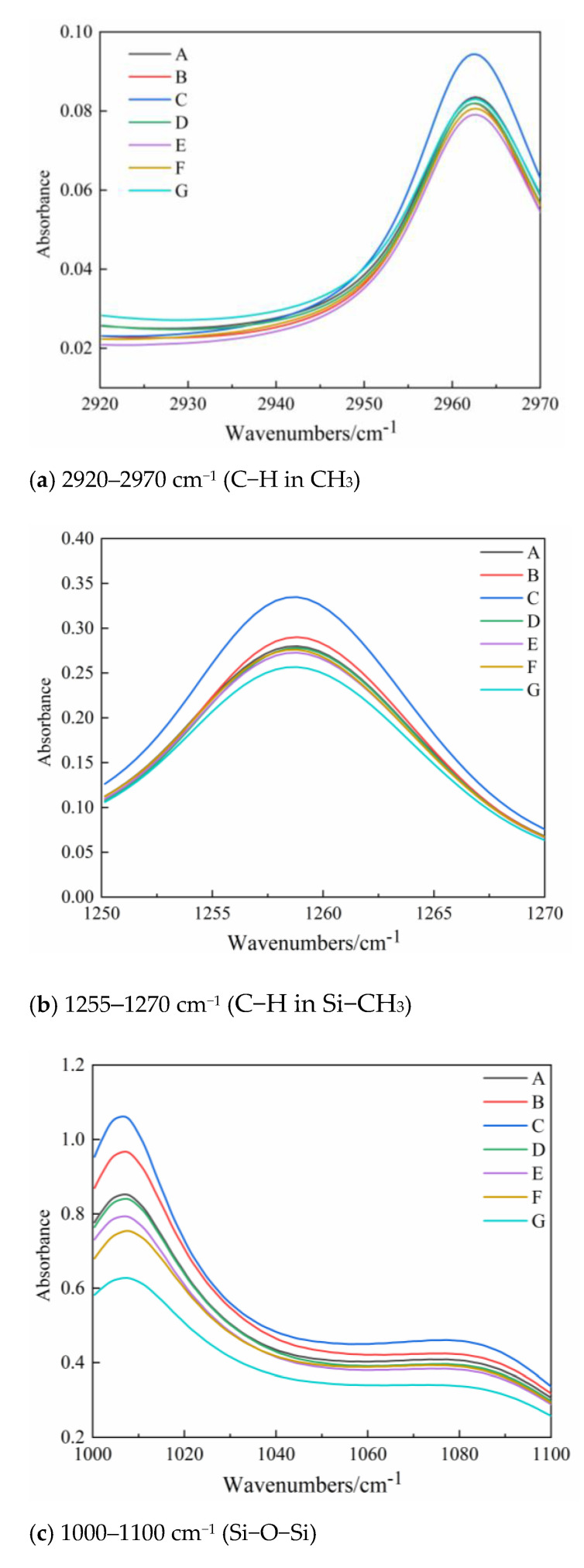

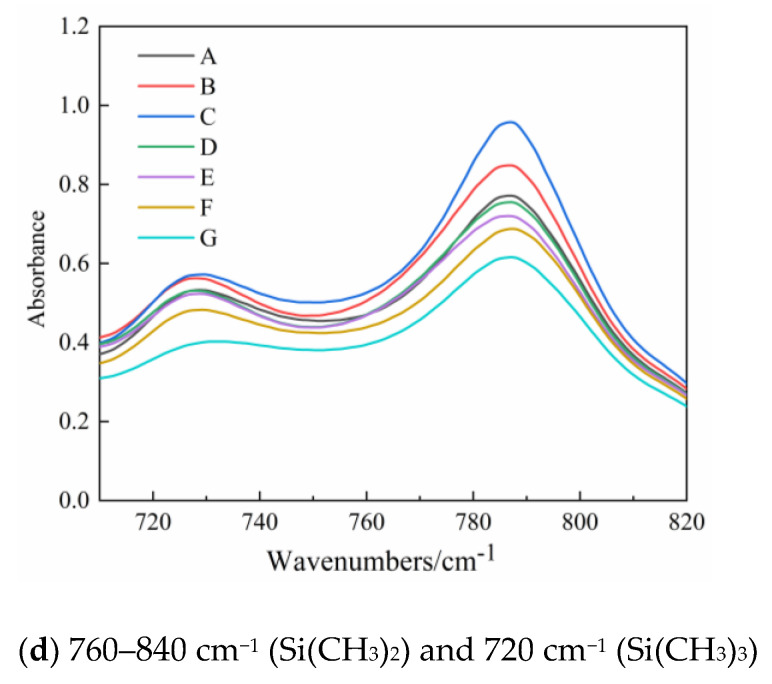
Infrared spectra of different wavenumber ranges.

Corresponding functional groups with wavenumbers of 2920–2970 cm^−1^, 1255–1270 cm^−1^, 1000–1100 cm^−1^, 760–840 cm^−1^, and 720 cm^−1^ are analyzed, as shown in Figure 10a–d, respectively.

Combined with the infrared radiation characteristic peaks of typical groups in Table 5, the absorption peaks of each functional group in Figure 10 are analyzed. It can be seen that: E > B > D > A > F > C > G.

(1) The wavenumber of 2920–2970 cm^−1^ indicates the existence of methyl (−CH_3_). As indicated in Figure 10a, the order of absorption peaks is C > A ≈ G > D ≈ B > F > E. The level of absorption peak represents the amount of methyl content.
  1# insulator has the highest absorption peak at the high voltage end. The absorption peak at three positions of 1# insulator is higher than that of 2# insulator. 2# insulator among the seven samples has the lowest absorption peak (in the middle). The decrease in C−H bond content was mainly due to the fracture of −CH_3_ functional groups in silicone rubber;
(2) The corresponding functional group at the wavenumber of 1255–1270 cm^−1^ is C−H in Si−CH_3_. As shown in Figure 10b, the order of absorption peaks is C > B > A > D > F > E > G. The absorption peak of the high voltage end of 1# insulator is the highest, indicating that the C−H bond in Si−CH_3_ in the high voltage end is the most. The absorption peak at three positions of 1# insulator is higher than that of 2# insulator. The peak value of the low voltage side of the two operating insulators is close, indicating that the aging degree is similar. Furthermore, 3# insulator’s absorption peak is lowest on the high voltage side.
  The decrease in −CH_3_ indicates that the macromolecular chain is broken, further weakening hydrophobicity, according to the findings shown in Table 3. In addition, the degree of damage caused by −CH_3_ can, to a certain extent, reflect the degree of aging. The Si−O−Si bond is broken, and small silicone molecules are lost, which reduces the absorption peak value;
(3) Figure 10c indicates that at the wavenumber of 1000–1100 cm^−1^, the corresponding functional group is Si−O−Si, and the change in the absorption peak is identical to that of methyl (−CH_3_);
(4) The two peaks in Figure 10d represent Si(CH_3_)_2_ (760–840 cm^−1^) and Si(CH_3_)_3_ (720 cm^−1^), respectively. It can be seen from Figure 10d absorption peak: C > B > A > D > E > F> G. The decrease of Si−(CH_3_)_2_ groups means that the silicone rubber is polarized, and the hydrophobicity decreases.

The sheds become harder as inorganic silicon precipitates from the silicone rubber surface. The results are consistent with the previously mentioned hardness and hydrophobicity. The comparison of 7 samples reveals the overall aging degree: 1# >2# > 3#. The aging degree at the high voltage end of 1# insulator is the most severe. Considering the decay-like fracture at the high voltage end, it is assumed that decay fracture is closely related to aging.

### 3.3. Preventive Measures

Literature [37] proposed the use of the nano modification method to improve the corrosion resistance of epoxy resin matrix in FRP, which has been studied in the field of nano dielectric (composite) materials [38,39]. In addition, many measures are proposed to reduce the occurrence probability of composite insulator decay fracture. As a new type of rigid composite insulator, the grease ring epoxy resin is used to replace the original silicone rubber sheath, which has the advantages of good electrical insulation and high mechanical strength [40,41,42].

## 4. Conclusions

The investifations of the visual, physical, and chemical properties of three insulators (decay-like fractured insulator, actual operating insulator on the V string, and new insulator) lead to the following conclusions, which are presented in this paper:

(1) The sheds’ degradation is concentrated on the side with heavy pollution, with no notable occurrence on the other side. Along the direction of the grounding end, the degree of degradation diminishes. After a long-term operation, due to the hydrophobic migration characteristics of silicone rubber, the two insulators still maintain good hydrophobicity. From the morphology, pollution characteristics, and hydrophobicity, the aging degree of the high voltage side is higher than that of other parts in the same insulator. There is no direct connection between the physical properties of sheds and decay-like fracture of the core rod;
(2) The severity of aging increases with a decrease in the equivalent transverse relaxation time T_2_. The main chain of PDMS is severely damaged at the insulator fracture. NMR and FTIR can well judge the aging degree of silicone rubber of composite insulators. However, the aging degree of silicone rubber cannot be used to judge whether the composite insulator is decay-like or level;
(3) By comparing the two insulators on the same V string, the physical and chemical properties of the fractured insulator sheds are poor, and the aging is serious, but no significant characteristics can characterize the difference between decay-like fractures. The operating instulators can also obtain the physical and chemical characteristics of the decay-like fractured insulator sheds after enough time, so it is difficult to judge the decay-like fracture only by the aging degree of the silicone rubber sheds.

## Figures and Tables

**Table 1:** Pollution test results.

Type	Sample	ESDD_top_/ESDD_bottom_^1^ (mg/cm^2^)	ESDD (mg/cm^2^)	NSDD_top_/NSDD_bottom_ ^2^ (mg/cm^2^)	NSDD (mg/cm^2^)
1#	A	0.042/0.025	0.022	1.926/0.114	0.497
	B	0.026/0.012	0.015	1.525/1.053	1.154
	C	0.047/0.015	0.029	1.690/1.440	1.493
2#	D	0.031/0.026	0.027	2.014/0.990	1.207
	E	0.032/0.011	0.015	2.430/1.039	1.335
	F	0.043/0.013	0.019	2.319/1.124	2.065

^1^ ESDD_top_ and ESDD_bottom_ refer to the ESDD of the sheds’ top and bottom surfaces, respectively. ^2^ NSDD_top_ and NSDD_bottom_ refer to the NSDD of the sheds’ top and bottom surfaces, respectively.

**Table 2:** The hardness (Shore A) of silicone rubber samples.

Type	Sample	K1	K2	K1−K2	(K1−K2)/K1
1#	A	68.4	66.2	2.2	3.22%
	B	69.2	65.0	4.2	6.07%
	C	75.4	73.2	2.2	2.92%
2#	D	69.4	66.4	3.0	4.32%
	E	71.0	70.8	0.2	0.28%
	F	75.4	73.4	2.0	2.65%
3#	G	66.2	64.8	1.4	2.11%

**Table 3:** The static contact angles of the samples.

Type	Sample	θ_AVG_ (°) ^1^	θ_MIN_ (°) ^2^
1#	A	143.720	139.680
	A *	123.735	120.180
	B	146.317	142.752
	B *	131.653	124.148
	C	146.347	141.923
	C *	104.986	102.728
2#	D	143.488	140.619
	D *	130.259	129.067
	E	148.592	147.275
	E *	134.458	134.054
	F	126.329	126.048
	F *	116.941	114.109
3#	G	101.751	100.742

^1^ θ_AVG_ refers to the average value of static contact angle. ^2^ θ _MIN_ refers to the minimum value of static contact angle. * refers to the sample after cleaning contamination.

**Table 4:** NMR test results.

Type	Sample	Average (ms)
1#	A	59.5717
	A *	57.0346
	B	60.0761
	B *	56.3128
	C	52.7898
	C *	64.0521
2#	D	59.6568
	D *	56.5934
	E	63.5008
	E *	58.0489
	F	56.1363
	F *	62.4736
3#	G	68.1456

* refers to the sample after cleaning contamination.

**Table 5:** Typical characteristic peaks of the silicone rubber and positions.

Wavenumbers/cm^−1^	Functional Groups
3200–3700	−OH
2920–2970	C−H in CH_3_
1255–1270	C−H in Si−CH_3_
1000–1100	Si−O−Si
760–840	Si(CH_3_)_2_
720	Si(CH_3_)_3_

## Data Availability

Data presented in this study are available on request from the first author.

## Abbreviations

FRP	Fiber Reinforced Plastics
FTIR	Fourier transform infrared spectroscopy
NMR	Nuclear Magnetic Resonance
HTV	High Temperature Vulcanization
SiR	Silicone Rubber
ESDD	Equivalent Salt Deposit Density
NSDD	No Soluble Deposit Density
PDMS	Polydimethylsiloxane
ATH	Alumina Tri-hydrate
CPMG	Carr−Purcell−Meiboom−Gill
